# Transiently Activated Human Regulatory T Cells Upregulate BCL-XL Expression and Acquire a Functional Advantage *in vivo*

**DOI:** 10.3389/fimmu.2019.00889

**Published:** 2019-04-24

**Authors:** Fadi Issa, Kate Milward, Ryoichi Goto, Gareth Betts, Kathryn J. Wood, Joanna Hester

**Affiliations:** Transplantation Research Immunology Group, Nuffield Department of Surgical Sciences, University of Oxford, Oxford, United Kingdom

**Keywords:** regulatory T cells, Tregs, tolerance, rejection, BCL-XL, *in vivo*, humanized mouse model, apoptosis

## Abstract

Regulatory T cells (Tregs) can control excessive or undesirable immune responses toward autoantigens, alloantigens, and pathogens. In transplantation, host immune responses against the allograft are suppressed through the use of immunosuppressive drugs, however this often results in life-threatening side effects including nephrotoxicity and an increased incidence of cancer and opportunistic infections. Tregs can control graft-vs.-host disease and transplant rejection in experimental models, providing impetus for the use of Tregs as a cellular therapy in clinical transplantation. One of the major barriers to the widespread use of Treg cellular therapy is the requirement to expand cells *ex vivo* to large numbers in order to alter the overall balance between regulatory and effector cells. Methods that enhance suppressive capacity thereby reducing the need for expansion are therefore of interest. Here, we have compared the function of freshly-isolated and *ex vivo*-manipulated human Tregs in a pre-clinical humanized mouse model of skin transplantation. Sorted human CD127^lo^CD25^+^CD4^+^ Tregs were assessed in three different conditions: freshly-isolated, following transient *in vitro* activation with antiCD3/antiCD28 beads or after *ex vivo*-expansion for 2 weeks in the presence of antiCD3/antiCD28 beads and recombinant human IL2. While *ex vivo*-expansion of human Tregs increased their suppressive function moderately, transient *in vitro*-activation of freshly isolated Tregs resulted in a powerful enhancement of Treg activity sufficient to promote long-term graft survival of all transplants *in vivo*. In order to investigate the mechanisms responsible for these effects, we measured the expression of Treg-associated markers and susceptibility to apoptosis in activated Tregs. Transiently activated Tregs displayed enhanced survival and proliferation *in vitro* and *in vivo*. On a molecular level, Treg activation resulted in an increased expression of anti-apoptotic *BCL2L1* (encoding BCL-XL) which may be at least partially responsible for the observed enhancement in function. Our results suggest that *in vitro* activation of human Tregs arms them with superior proliferative and survival abilities, enabling them to more effectively control alloresponses. Importantly, this transient activation results in a rapid functional enhancement of freshly-isolated Tregs, thereby providing an opportunity to eliminate the need for *in vitro* expansion in select circumstances. A protocol employing this technique would therefore benefit from a reduced requirement for large cell numbers for effective therapy.

## Introduction

Regulatory T cells (Tregs) are critical in the control of immune homeostasis as demonstrated by the development of autoimmune pathologies following their elimination (1) and the resolution of disease following their adoptive transfer (2). However, due to the high precursor frequency of alloreactive T cells in transplantation, unless the balance of Tregs to T effector cells (Teffs) is significantly altered, transplants are rejected despite the presence of functional Tregs (3). Current clinical practice is focused mainly on disarming the effector arm of the alloresponse using immunosuppressive drugs to deplete T cells or inhibit their proliferation and function (4). Such an approach leads to generalized immunosuppression, exposing patients to the cytotoxic effects of these drugs and increasing the risk of cancer and opportunistic infections. There is therefore increasing focus on studying the biology and function of Tregs for their ability to control graft-vs.-host disease (GVHD) and allograft rejection with the aim of utilizing them as a cellular therapy (5, 6).

Tregs are commonly described as CD25^+^CD4^+^ T cells in mice and CD127^lo^CD25^+^CD4^+^ T cells in humans, and exhibit sustained expression of the master regulator transcription factor, FOXP3. Thymically-derived, naturally-occurring Tregs, tTregs, are the population most studied as a possible source of therapeutic cells, with the majority of protocols utilizing *in vitro* expanded tTregs [reviewed in (7, 8)]. So far, both freshly isolated (9) and *in vitro* expanded tTregs (10) have been tested in phase I clinical trials as a prevention of GVHD after HSC transplantation and proved to be safe, however their comparative efficacy is unclear and has not been tested so far.

Humanized mouse models provide a useful pre-clinical tool to study *in vivo* effectiveness of human Treg populations. Using these models, expanded human CD127^lo^CD25^+^CD4^+^ Tregs have been shown to control rejection in vessel (11), islet (12) and skin (13, 14) transplantation and to prevent GvHD (15). However, the direct comparison of the *in vivo* potency of freshly isolated and *in vitro* expanded human Tregs is lacking. In this study, we compare the ability of suboptimal doses of freshly sorted and *in vitro* expanded human CD127^lo^CD25^+^CD4^+^ Tregs to promote human skin allograft survival and demonstrate that higher effectiveness of expanded Tregs can be compensated by transient activation of freshly isolated Tregs. Recently-activated Tregs are characterized by an increased expression of Treg functional markers and better *in vitro* and *in vivo* survival, correlating with an increased expression of anti-apoptotic BCL-XL. The ability to enhance Treg function without long *in vitro* culture may be of value in the treatment of specific immunopathological situations.

## Materials and Methods

### Mice

Immunodeficient BALB/c Rag2^−/−^ IL2rγ^−/−^ mice were purchased from Jackson Laboratories (Maine, USA) and housed under specific pathogen-free conditions in the Biomedical Services Unit at the John Radcliffe Hospital (Oxford, UK). Animals were treated with strict accordance to the UK Animals (Scientific Procedures) Act of 1986 and under PPL P8869535A. Mice between ages of 6 and 12 weeks were used.

### Procurement of Human Skin and Blood

Healthy skin and blood was donated from patients undergoing plastic surgery procedures as previously described (13) and with full informed consent under approval number 07/H0605/130 from the Oxfordshire Research Ethics Committee B. PBMCs were isolated from buffy coats or leukocyte cones from healthy volunteers (NHSBT, UK).

### Skin Grafting

Skin grafting was performed as previously described (13). Briefly, 1 × 1-cm piece of human skin was fashioned and sutured to the mouse recipient skin on the left dorsal thorax over the costal margin. Grafts were left to heal for 35 days, before receiving an intraperitoneal injection of 5 × 10^6^ human peripheral blood mononuclear cells (PBMCs) allogeneic to the graft donor. Skin grafts were monitored regularly until loss. In experimental groups with Treg cells, 1 × 10^6^ Tregs from the same donor as PBMCs were coinjected with PBMCs. In all mice the degree of human leukocyte reconstitution was measured by flow cytometry at the time of harvest. Mice with human leukocyte chimerism levels of >0.1% in the blood or >1% in the spleen were defined as reconstituted and included in the study (13). Skin allograft survival time was calculated from the point of PBMC injection to the point of complete graft loss/visible rejection.

### Sorting and Expansion of Human Tregs Cells

Human Tregs were sorted and expanded as previously described (16) with minor modifications. Briefly, CD25^+^ cells were bead-enriched (CD25 Microbeads, Miltenyi Biotech) from PBMCs isolated from buffy coats from healthy volunteers (NHSBT, UK). CD127^lo^CD25^+^CD4^+^ Tregs were sorted using a BD FACSAria cell sorter (Becton Dickinson) after staining with anti-CD127 PE, anti-CD25 PE-Cy7 (both Becton Dickinson) and anti-CD4 ECD (Beckman Coulter). Sorted cells were either used unmanipulated, activated overnight (15 h in 37°C 5%CO_2_ with anti-CD3/anti-CD28 beads (Invitrogen) at 1:5 bead:cell ratio), or expanded *in vitro* with 1000U/ml recombinant human IL-2 (rhIL-2, Chiron) and anti-CD3/anti-CD28 beads (Invitrogen) during two, 7 days long, expansion rounds, followed by resting after beads removal. Cells were cultured in RPMI-1640 medium (Sigma) supplemented with L-glutamine, penicillin-streptomycin (both Sigma), sodium pyruvate (Gibco) and 10% human AB pooled serum. In some experiments, expanded Tregs were activated overnight in a similar manner to sorted Treg (15h in 37°C 5%CO_2_ with anti-CD3/anti-CD28 beads at 1:5 bead:cell ratio).

### *In vitro* Suppression Tests

To assess Treg suppressive capacity, 5 × 10^4^ PBMC were incubated with 1 × 10^5^ irradiated allogeneic PBMC and co-cultured with decreasing number of Tregs. ^3^H-thymidine (Perkin Elmer) was added for the last 16–18 h of the 7 day culture. All experimental conditions were done in 4–6 replicates. Results were obtained as cpm (counts per minute) and normalized to positive control (PBMC+allo) with positive control values setup as maximum (100%) proliferation and all other values recalculated accordingly. Cpm values over 10,000 for positive control (PBMC+allo) were required to classify test as passing quality control for proliferation.

### *In vitro* Cellular Proliferation and Apoptosis Assays

To measure proliferation and apoptosis, freshly isolated Tregs were stained with 10 μM Cell Trace Violet (CTV; Invitrogen), activated for 15 h with anti-CD3/anti-CD28 beads (at a ratio of 1 bead to 5 cells ratio) or left untreated. After activation, beads were carefully removed and cells plated at 10^5^ onto 96 well U bottom plates in the presence or absence of rhIL-2 at 250 U/ml. After 5 days of culture, cells were washed with Annexin V binding buffer and stained with Annexin V and 7-AAD (both eBioscience). Stained cells were resuspended in Annexin V binding buffer and FACS analyzed within an hour.

### *In vivo* Survival and Proliferation

To measure *in vivo* survival and proliferation, freshly isolated Tregs were stained with 10 μM CTV (Invitrogen), activated for 15 h with anti-CD3/anti-CD28 beads (at a ratio of 1 bead to 5 cells) or left untreated. After activation, beads were carefully removed and 1 × 10^6^ Treg cells were mixed with 5 × 10^6^ PBMCs, labeled with 1 μM CFSE (eBioscience), from the same donor. PBMCs and Tregs were injected intraperitoneally into BALB/c Rag2^−/−^ IL2rγ^−/−^ mice and human cells isolated from peritoneal lavage on d5 for analysis as previously described (17).

### Flow Cytometry

For analysis of *in vivo* experiments, cells were stained with antibodies against human CD45 APC (Invitrogen), CD4 ECD (Beckman Coulter), CD3 Pacific Blue (eBioscience) CD8 PE and CD25 PE-Cy7 (BD) and the viability dye 7-AAD (eBioscience). To analyse expression of Treg-associated markers, freshly sorted or expanded Tregs were stimulated for 15h with anti-CD3/anti-CD28 beads at a ratio of 1 bead to 5 cells, or left untreated. Cells were stained with 7-AAD and antibodies against GITR FITC (R&D Systems), CTLA-4 PE, CD69 APC-Cy7, CD25 PE-Cy7 (BD), TIGIT PE, OX-40 FITC, TIM-3 APC, CD39 PE, FOXP3 eFluor 450, Perforin APC (eBioscience) Helios AlexaFluor 647 (Biolegend), and CD4 ECD (Beckman Coulter). For intracellular antigens (FOXP3, Helios, Perforin, CTLA-4), cells were fixed and permeabilized using a Foxp3 Staining Buffer Set (eBioscience). Samples were acquired using a BD FACSCanto (BD Biosciences) and analyzed using FACSDiva software (BD Biosciences). For staining for BCL-XL and MCL1, Abcam anti-BCL-XL FITC (7B2.5; ab26148) and anti-MCL-1 Alexa Fluor 488 (Y37, ab197529) antibodies were used, respectively, following manufacturer's instructions. Briefly, the cells were fixed with 4% paraformaldehyde and permeablized with PBS/0.1% Tween. The cells were then blocked with 10% normal goat serum/0.3 M glycine, followed by incubation with the antibody.

### Real-Time PCR

Total RNA (including small RNA) was isolated from cell pellets using a mirVana PARIS Kit (Ambion, Applied Biosystems). For mRNA analysis, cDNA was generated with High Capacity RNA-to-cDNA Kit (Applied Biosystems) followed by real-time PCR using *HPRT* primers and probe as described previously (18) and TaqMan Gene Expression Assays (Applied Biosystems) for the following genes: Hs99999146_m1 (*BCL2L*1, coding BCL-XL), Hs00608023_m1 (*BCL2*), Hs99999001_m1 (*BAX*) and Hs01083836_m1 (*BCL2L11*, coding BIM). Stratagene Mx3000P thermo cycler (Agilent Technologies) was used for qPCR. Delta Ct values were calculated using *HPRT* as an endogenous control and converted to 2^−deltaCt^ values.

For microRNA analysis, samples were analyzed for expression of RNU48 (assay ID 001006), hsa-miR-16-5p (assay ID 000391) and hsa-let-7c (assay ID 000379) using TaqMan MicroRNA Assays (Life Technologies). cDNA reactions were performed using TaqMan MicroRNA Reverse Transcription Kit (Life Technologies). Subsequent qPCR was performed using TaqMan Universal Master Mix II and MicroRNA Assays using a Stratagene Mx3000P thermo cycler (Agilent Technologies). Delta Ct values were calculated using RNU48 as an endogenous control and converted to 2^−deltaCt^ values.

### Tissue Typing

Blood from buffy coats and skin donors was analyzed at the Oxford Transplant Center Transplant Immunology and Immunogenetics laboratory. A full typing was performed for HLA-A, -B, -Cw, -DR and -DQ using PCR-SSP method.

### Statistical Analysis

Statistical evaluations were performed using Graphpad Prism software (GraphPad Software Inc, California). Survival data were analyzed using log-rank tests. Groups of three or more were compared using a non-parametric Kruskal-Wallis ANOVA with Dunn's *post-hoc* multiple comparisons test. For comparison of two groups non-parametric Mann-Whitney *U*-tests were applied. To assess differences between *in vitro* suppressive capacities of different Treg populations the area under the curve (AUC) method was used as described by previously (19). *p*-values < 0.05 were considered significant.

## Results

### *Ex vivo* Expanded Human Tregs Cells Have Increased *in vitro* and *in vivo* Suppressive Capacity

**Figure 1:**
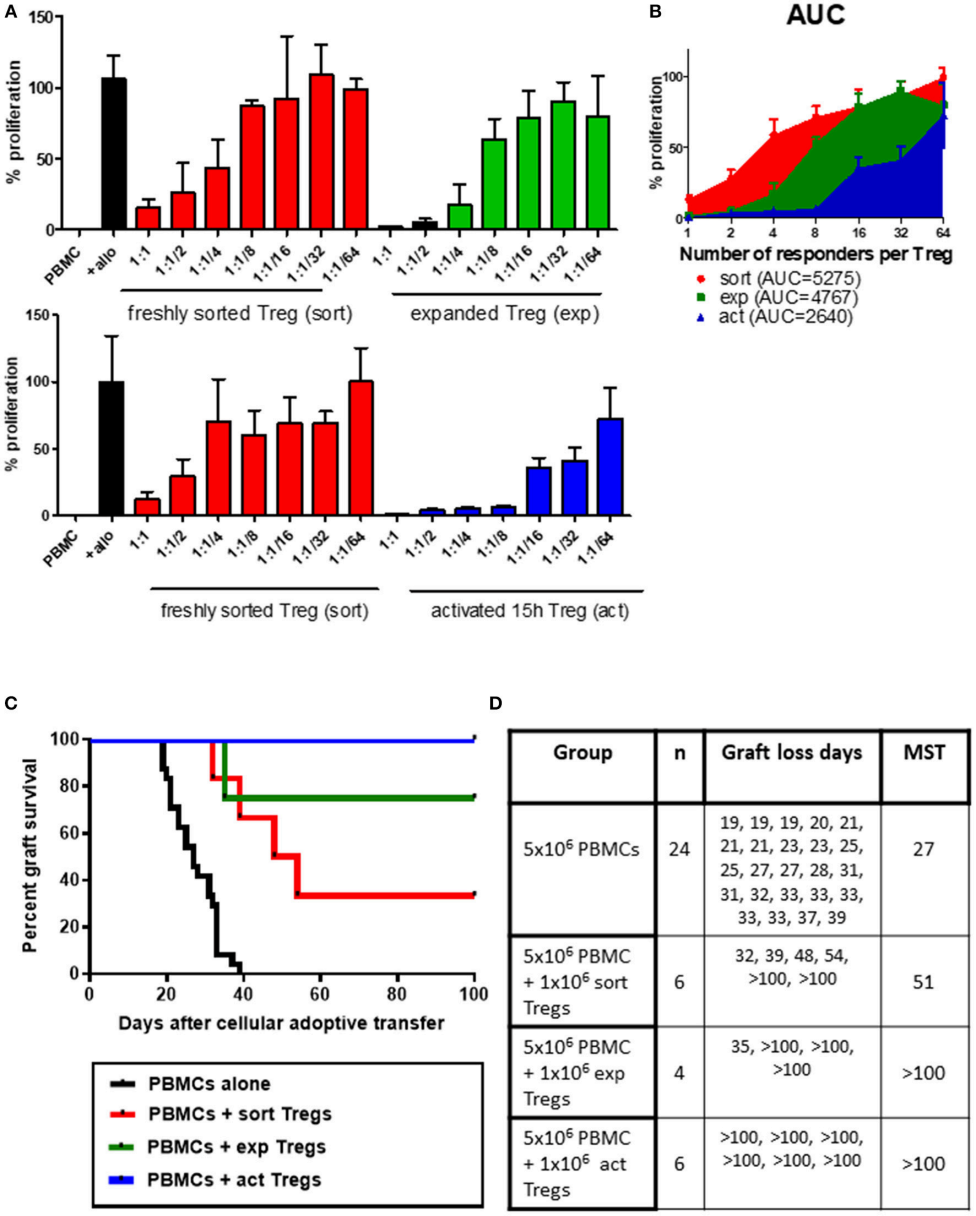
*Ex vivo* expanded or transiently activated human Tregs have superior *in vitro* and *in vivo* suppressive capacity. **(A)** 5 × 10^4^ PBMCs were incubated with 1 × 10^5^ irradiated allogeneic PBMCs and co-cultured with a decreasing number of freshly isolated (sort) or *ex vivo* expanded (exp) (top panel) or transiently activated (act) (bottom panel) human Tregs. Ratios represent ratio of responder PBMC to Tregs. Data represented as percentage of proliferation of allogeneically-stimulated PBMC. **(B)** Area under curve for the frequency of proliferating responders in the presence of Tregs at different ratios. **(C)** Mice received a skin graft and 5 × 10^6^ allogeneic PBMCs together with 1 × 10^6^ freshly isolated, transiently activated (15 h) or *ex vivo* expanded Tregs. Data are pooled from different skin/cell donor combinations. **(D)** Table representing data from B, MST—median survival time. *p*-values using the log-rank test for each comparison are shown.

The main aim of expanding Tregs *ex vivo* is to provide a sufficient number of cells for clinical application, altering the balance between Tregs and Teffs (20). However, the process of expansion has the potential to impact Treg biology and function. To determine whether expansion of Tregs influences their suppressive function, Tregs were sorted from a single donor, rested overnight and assessed directly in a suppression test, or *in vitro* expanded in the presence of anti-CD3/anti-CD28 beads and rhIL-2 before also being rested and then subjected to the same suppression assay assessment. At the higher Treg to responder ratios, expanded Tregs have shown a trend toward higher suppressive capacity than freshly sorted Tregs (Figure 1A top panel, Figure 1B). We next assessed whether this observation was also reflected *in vivo*. We have previously shown that treatment with *ex vivo* expanded Tregs results in long-term graft acceptance when adoptively transferred at a ratio of 1:1 Tregs to PBMCs (13), whereas a reduced (or “suboptimal”) number of Tregs (1:5 Tregs:Teff or below) results in graft prolongation (21). To assess the *in vivo* suppressive capacities of freshly isolated and *ex vivo* expanded Tregs, each of these populations was adoptively transferred together with PBMCs at the suboptimal 1:5 dose. As expected, expanded Tregs provided significant allograft prolongation with 75% grafts being accepted long-term (Figures 1C,D, median survival time (MST)>100 days, *p* = 0.0013 vs. PBMC group). However, in line with the *in vitro* data, freshly isolated Tregs only temporarily extended graft survival (MST 51 days compared to 27 days in the PBMC alone group) with 2 out of 6 grafts accepted long-term (Figures 1C,D, *p* < 0.0001 vs. PBMC group).

### Transient Activation Increases Suppressive Abilities of Freshly Isolated Tregs

Having observed trend toward enhanced suppressive capacity of expanded human Tregs over their freshly isolated counterparts, we next asked whether *ex vivo* expansion is necessary for Tregs to increase their regulatory capacity or whether activation without expansion would be sufficient. We therefore activated freshly isolated human Tregs for 15 h in the presence of anti-CD3/anti-CD28 beads and compared their suppression *in vitro* to non-activated freshly isolated Tregs. This transient activation notably increased the regulatory capacity of Tregs, an effect which was especially pronounced at higher Treg to PBMC ratios (Figure 1A bottom panel, Figure 1B). In support of the *in vitro* suppression data, transient anti-CD3/anti-CD28 activation of Tregs also resulted in excellent regulation of alloresponses *in vivo*, leading to long-term acceptance of all skin allografts (Figures 1C,D, MST >100 days, *p* < 0.0001 vs. PBMC group). Transiently activated Tregs were more effective *in vivo* than freshly isolated cells (*p* = 0.0137), however there was no statistically significant difference between *in vivo* function of short-term activated and expanded Tregs (*p* = 0.2207).

### *In vitro* Activation Increases Expression of Treg Markers

**Figure 2:**
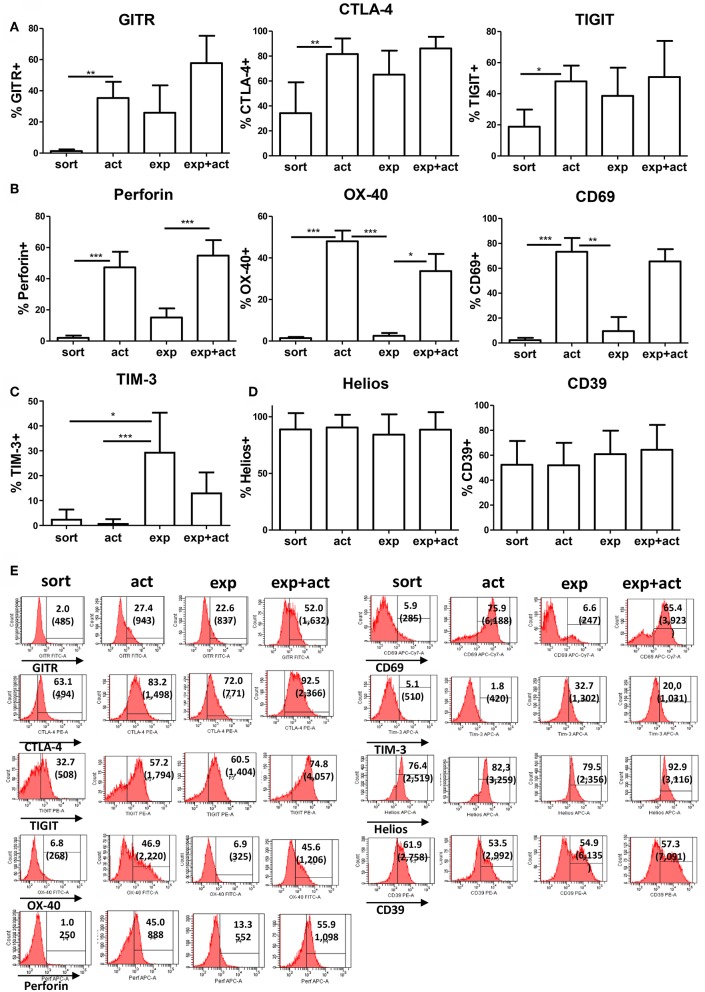
Expression of Treg-associated markers. **(A–D)** Freshly isolated (sort), transiently activated (act), *ex vivo* expanded (exp) and transiently reactivated expanded (exp+act) human Tregs were stained for expression of Treg-associated markers. **(E)** Representative FACS plots of the Treg-associated markers shown in **(A–D)**. *n* = 4–7 separate donors. *p < 0.05 Error bars represent the means and standard deviations (SD). Groups were compared using a non-parametric Kruskal-Wallis ANOVA with Dunn's *post-hoc* multiple comparisons test. **p < 0.01, ***p < 0.001.

**Figure 3:**
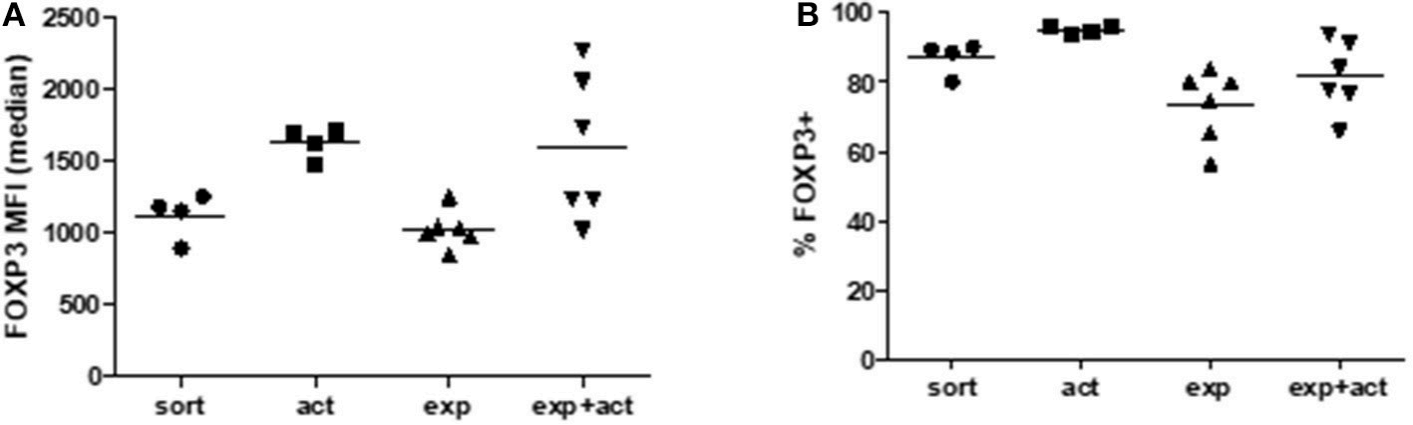
FOXP3 expression in Tregs after activation or expansion. Freshly isolated (sort), transiently activated (act), *ex vivo* expanded (exp) and transiently reactivated expanded (exp+act) human Tregs were stained for expression of FOXP3 and both **(A)** Median fluorescence intensity (MFI) and **(B)** percentage expression of FOXP3 were both measured. *n* = 4–6 separate donors. Lines indicate mean values. Groups were compared using a non-parametric Kruskal-Wallis ANOVA with Dunn's *post-hoc* multiple comparisons test.

Next, we examined the expression of Treg-associated markers in Tregs either following activation for 15 h, after *in vitro* expansion, or immediately following fresh isolation (Figure 2). An additional group of transiently reactivated expanded Tregs was also examined to determine whether restimulation following expansion promotes the upregulation of the same molecules as in stimulated freshly isolated cells. The classical Treg markers GITR and CTLA-4 were up-regulated both after transient activation and *in vitro* expansion as compared with freshly isolated Tregs, although the difference was only statistically significant for transient activation (Figures 2A,E). A similar pattern of expression was also observed for the Ig family member with immunomodulatory function, TIGIT (22, 23) (Figures 2A,E). As expected, the early T cell activation marker, CD69, was induced upon activation (Figures 2B,E). Similarly, OX-40 and perforin expression was induced on transiently activated Tregs, both freshly isolated and expanded with statistically significant differences observed for both markers, both after transient activation of freshly isolated cells and restimulation post-expansion (Figures 2B,E), suggesting different expression kinetics compared with GITR, CTLA-4 and TIGIT. T cell immunoglobulin and mucin domain-3 protein (TIM-3), which is expressed on fully differentiated effector T cells [mainly Th1 (24) and Th17 (25) cells], and was described as a marker of short-lived but highly suppressive Tregs (26) was expressed by about 20–30% of expanded Treg but not by freshly isolated or transiently activated Tregs (Figures 2C,E). In contrast, expression of CD39 and Helios was not changed upon activation or expansion (Figures 2D,E). There was a trend toward an increase in FOXP3 expression levels per cell after activation, although this was not statistically significant (Figure 3A). The proportion of FOXP3+ cells was also not different after activation (Figure 3B). Overall, the phenotypic analysis of *in vitro* stimulated Tregs demonstrated significant differences in expression of functional Treg markers, in agreement with their increased suppressive function.

### Transient Activation Promotes Treg Proliferation and Survival

**Figure 4:**
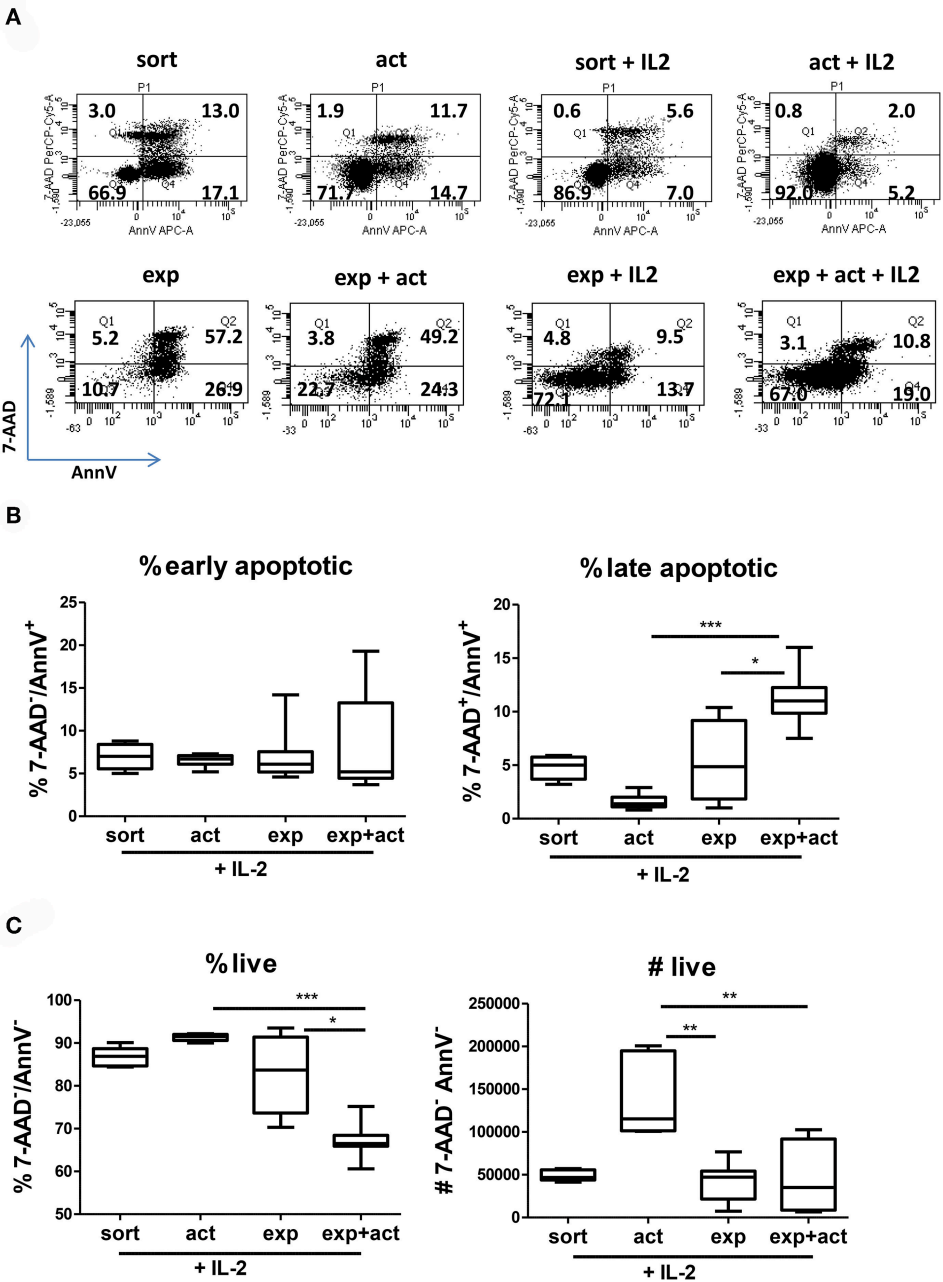
Transient activation promotes Treg survival and responsiveness to IL-2. **(A–C)** CTV-labeled Tregs (10^5^ per well) were activated *in vitro* for 15 h with anti-CD3/anti-CD28 beads and cultured for 5 days without further stimulation or cultured without any TCR stimulation at all. Where indicated (+IL-2), exogenous IL-2 was added. **(A)** Representative dot plots of cells stained with 7-AAD and Annexin V and the percentage of cells in each quadrant are depicted. **(B)** Percentage of early apoptotic (7-AAD^−^/AnnV^+^), late apoptotic (7-ADD^+^/AnnV^+^) and **(C)** percentage and number of live cells (7-AAD^−^/AnnV^−^) are shown. Data for **(A–C)** were obtained from 2 or 3 separate donors, each donor with 2–5 biological repeats (separate wells). Data from multiple cell donors were pooled. **(B,C)** min-max with median and interquartile range is shown. Groups were compared using a non-parametric Kruskal-Wallis ANOVA with Dunn's *post-hoc* multiple comparisons test. *p < 0.05, **p < 0.01, ***p < 0.001.

As demonstrated above, short-term transient activation and, to a certain degree, 2-week *in vitro* expansion of human Tregs influence the function of freshly isolated cells (Figure 1). The improved suppressive activity of both activated and expanded Tregs correlates with increased expression of Treg markers and functional molecules (Figure 3), however their differential expression pattern suggests further differences between both groups. To further explore the differences between freshly isolated and activated or expanded Tregs, we investigated the ability of Tregs to survive *in vitro* and *in vivo*. First, we measured the effect of transient activation on apoptosis of freshly isolated and expanded Tregs cultured for 5 days in the presence or absence of IL-2 without further stimulation. Without IL-2, the majority of expanded Tregs and about 30% of freshly isolated Tregs underwent apoptosis (Figure 4A, left panels), whereas the addition of exogenous IL-2 decreased the frequency of cells with apoptotic features (Figure 4A, right panels). More detailed analysis of cells undergoing apoptosis in the presence of IL-2, which is a major survival factor for Tregs (27), revealed that the frequency of late apoptotic (7-AAD^+^AnnV^+^) cells was decreased in activated freshly isolated Tregs but increased in activated expanded ones (Figure 4B). In line with this finding, the percentage of live cells (7-AAD^−^AnnV^−^) increased in stimulated freshly isolated cells but was reduced in activated expanded Tregs (Figure 4C). Importantly, when live cells were enumerated, there was a more than two-fold increase in cell number in activated freshly sorted Tregs as compared with expanded Tregs (Figure 4C, right panel).

**Figure 5:**
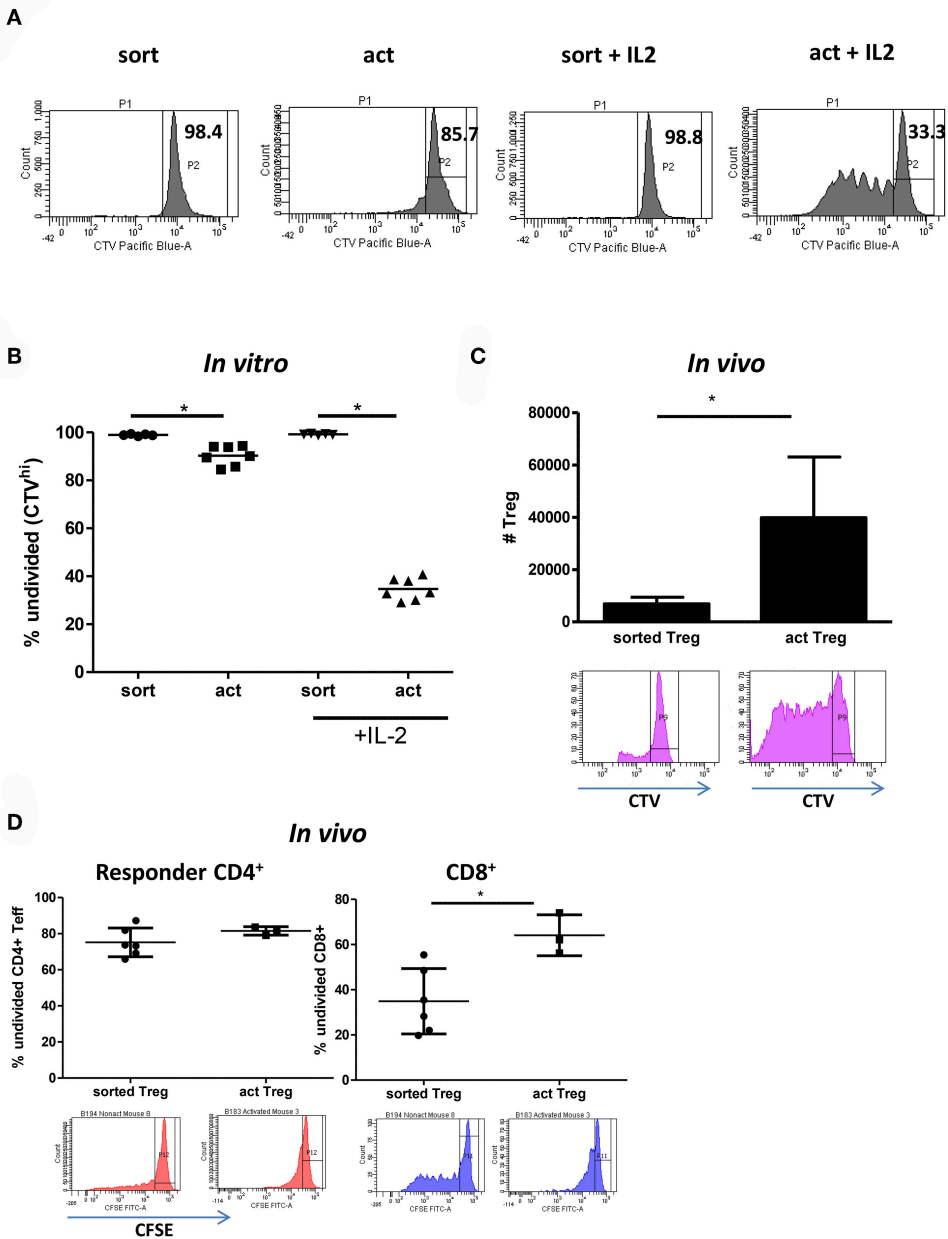
Transient activation is sufficient to induce Treg proliferation if IL-2 is present. **(A)** Representative histograms and **(B)** graph depicting the percentage of undivided (CTV^hi^) Tregs is shown. **(C)** 5 × 10^6^ CFSE-labeled PBMC and 1 × 10^6^ CTV-labeled freshly isolated Tregs (*n* = 6) or transiently activated Tregs (*n* = 3) were injected intraperitoneally into immunodeficient BRG mice. The number of CTV^+^ cells in the peritoneum on day 5 is depicted on the graph. Histograms represent CTV dilution in gated Tregs from freshly isolated and transiently activated Treg groups respectively. **(D)** Cells were prepared and injected as in **(C)**. The percentage of undivided (CFSE^hi^) responder CD4^+^ and CD8^+^ lymphocytes is shown. CFSE dilution in gated responder CD4^+^ and CD8^+^ T cells in mice receiving freshly isolated and transiently activated Tregs is shown. **(C)** mean with SD is depicted, **(D)** each point represents separate mouse. Groups were compared using a non-parametric Mann-Whitney *U*-test. *p < 0.05.

The 2–3 fold increase in the number but not percentage of live cells in activated Tregs suggested intense proliferation. Indeed, staining with the proliferation dye CTV revealed that close to 70% of activated Tregs activated with IL-2 divided at least once, with up to 6 divisions observed (Figure 5A). This was despite the stimulatory signal being removed after 15h. Conversely, unstimulated Tregs remained mainly undivided (Figure 5B). Next, we assessed whether activated Tregs are more likely to survive and expand *in vivo* by co-injecting BALB/c Rag2^−/−^IL2rγ^−/−^ mice with 5 × 10^6^ CFSE-labeled PBMCs and 1 × 10^6^ CTV-labeled Tregs and examining the number of CTV^+^ cells recovered in a peritoneal lavage after 5 days. In agreement with the *in vitro* data, we observed more Tregs in the activated group, with these cells displaying enhanced proliferation (Figure 5C). Importantly, activated Tregs were also found to be more suppressive *in vivo* than freshly isolated cells, as demonstrated by their ability to inhibit proliferation of CD8^+^ T cells in the co-injected PBMCs (Figure 5D).

### Activation Enhances Tregs *BCL2L1* (*BCL-XL*) Expression

**Figure 6:**
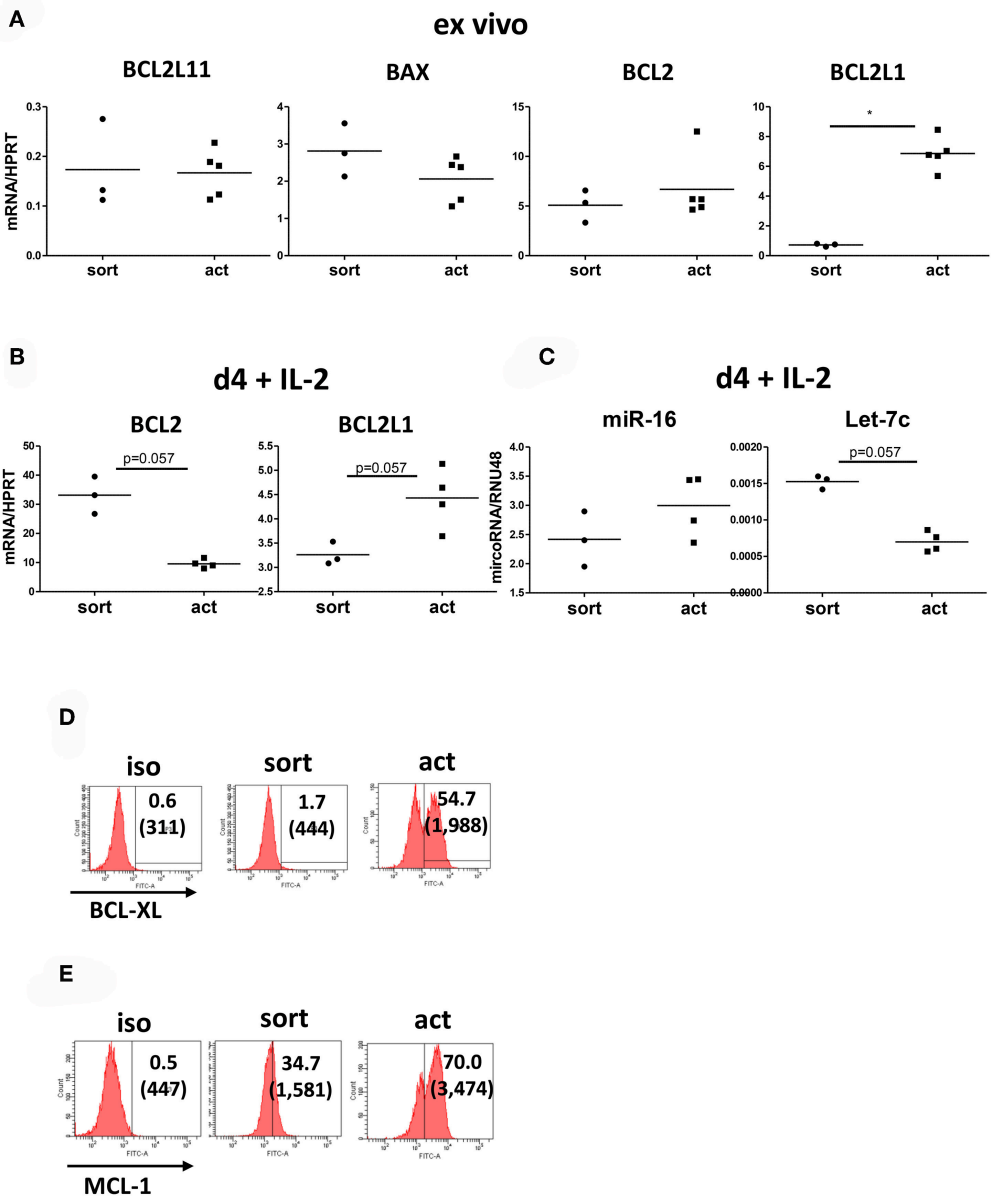
Transiently activated Treg upregulate *BCL2L1* (*BCL-XL*) expression. **(A)** Real-time PCR analysis of expression of genes encoding pro-apototic [*BAX, BCL2L11* (*BIM*)] and anti-apoptotic [*BCL2, BCL2L1 (BCL-XL)*] proteins in freshly isolated and transiently activated (15 h) Tregs. *n* = 3–5, data normalized to *HPRT*. **(B)** Real-time PCR analysis of *BCL2* and *BCL2L1* (*BCL-XL*) expression, normalized to *HPRT*, from day 4 cultures of unstimulated and 15 h transiently activated Tregs incubated with IL-2. **(C)** Real-time PCR analysis of miR-16 and Let-7c microRNA expression, normalized to RNU48, from cultures described in **(B)**. *n* = 3–4 donors, groups were compared using a non-parametric Mann-Whitney *U*-test, *p < 0.05. Additionally, levels of **(D)** BCL-XL and **(E)** MCL-1 were measured by FACS. Histograms from one representative donor are shown. *p < 0.05.

To further explore the finding that the transient activation of Tregs results in their enhanced survival, we measured the expression of pro-apoptotic [*BAX* and *BCL2L11* (encoding BIM)] and anti-apoptotic [*BCL2* and *BCL2L1* (coding BCL-XL)] genes in activated Tregs. Our real-time PCR data showed that *BCL2L1* but not *BLC2* expression was upregulated upon stimulation (Figure 6A). At the same time there was no difference in the expression of pro-apoptotic *BAX* and *BCL2L11* (Figure 6A), confirming that activated Tregs are less prone to apoptosis. Interestingly, when we incubated Tregs in the presence of IL-2 for 4 further days after removal of the activation stimulus, there was a trend toward upregulated expression of *BCL2L* and downregulated expression of *BCL2* when compared with unstimulated Tregs (Figure 6B). In order to further explore the possible mechanisms regulating *BCL2* and *BCL2L1* expression in activated Tregs, we measured the expression of miR-16 and Let-7c microRNAs, which have been implicated in the regulation of BCL2 (28) and BCL-XL (29, 30), respectively. Let-7c has been demonstrated to regulate BCL-XL expression in human hepatocellular carcinoma (30) and endothelial cells (29). Activated Tregs downregulated Let-7c expression (Figure 6C) while upregulating *BCL2L1* (BCL-XL) (Figure 6B), suggesting that Let-7c may also be engaged in the control of BCL-XL expression in Tregs. Conversely, there was no difference in miR-16 expression after Tregs activation (Figure 6C). Gene expression levels of *BCL2L1* correlated with increased BCL-XL levels on activated Tregs by FACS (Figure 6D). Levels of another apoptosis regulator, MCL1, were also increased (Figure 6E).

## Discussion

In recent years, the potential clinical application of Tregs both in transplantation and autoimmunity has attracted a great deal of enthusiasm. Here, we describe a feasible method for significantly enhancing human Treg suppressive capacity and demonstrate the efficacy of this approach in a pre-clinical *in vivo* transplantation model. We demonstrate that transient (15 h) *in vitro* activation of freshly isolated human Tregs is sufficient to provide a functional advantage over unmanipulated freshly isolated Tregs and may therefore eliminate the need to expand Tregs *in vitro* to achieve clinical efficacy. This provides a useful optimization technique for current protocols that employ Tregs as a cellular therapy to prevent GVHD or graft rejection [reviewed in (5, 6, 31)]. This may be of particular importance in clinical situations where expansion is impractical or where time restrictions on cellular manipulation exist.

There is no consensus at present as to whether freshly isolated or expanded Tregs should be used in clinical cellular therapy; each option having its distinct advantages. Freshly isolated Tregs were used by Martelli and colleagues as a strategy for GVHD prevention after HSC transplantation with no adverse effects and moderate clinical efficacy (9). Meanwhile, Blazar and his team used cord blood-derived expanded Tregs (10), again with no adverse effects and a reduction in the incidence of higher grade acute GVHD. Other groups have utilized expanded Tregs in T1D (32, 33) and kidney transplantation (34), whilst others yet have generated the cells from whole lymphocyte populations *ex vivo* (35). The advantage of using expanded Tregs is the ability to obtain large numbers of cells, however *ex vivo* expansion can be restrictively expensive and creates legitimate concerns regarding the safety of the clinical product as cells are subjected to a relatively long period (at least 2 weeks and in most cases 4–6 weeks) of *in vitro* culture and manipulation. From this aspect, the possibility to activate freshly isolated Tregs overnight before use as a clinical product may be particularly helpful in situations where obtaining relatively large numbers of non-expanded Tregs is feasible, such as in HSC transplantation where donor Tregs may be isolated from a leukapheresis product. Other uses include situations in which treatment with the regulatory cellular therapy must be instigated promptly and without prior notice, for example during an acute and unexpected autoimmune flare-up.

It is widely accepted that Tregs need to be activated by interaction of their TCR receptor with antigen presented in the context of MHC in order to elicit their suppressive ability. Additionally, it has been suggested that expanded Tregs have superior *in vitro* suppressive function compared with freshly isolated cells (36, 37). Indeed, in our study *ex vivo* expanded Tregs showed a tendency toward being more suppressive than freshly isolated cells. However, simply activating freshly isolated Tregs for 15 h increased their suppressive capacity significantly. This finding was supported by the observation that freshly isolated Tregs are less prone to apoptosis; a feature that is even more pronounced after transient activation.

While improvements in Treg survival are important, it is clear that this is unlikely to be the only mechanism accounting for the functional changes observed. Both transiently activated and expanded (but not activated) Tregs upregulate the expression of a number of Treg-associated markers including CTLA-4, GITR, TIGIT, and to a lesser degree, perforin. TCR activating signals are clearly important for Treg function, yet the duration and strength of these signals may result in different outcomes (38). Tregs likely require progressive signals provided at the appropriate time, to ideally result in enhanced survival and subsequent effective suppressive activity (39). Transiently activated Tregs may be at the first step of this sequential process. We also show that OX40 is induced on transient activation of Tregs but not expanded Tregs. The effects of OX40 expression or ligation on Tregs are complex and not completely understood (40). Some studies have demonstrated that OX40 stimulation can negatively regulate the induction of Tregs from naïve or effector T cells (41, 42), yet OX40 signaling has also been shown to be important in the generation of Tregs during the TCR-independent phase of Treg development, with OX40^−/−^ mice having significantly reduced numbers of Tregs (43). Given the potential for OX40 to be a therapeutic target, its function on T cells and Tregs remains under intense investigation.

Apoptosis is regulated through a balance between the Bcl-2 family of proteins, such as pro-apoptotic BAX and BIM and anti-apoptotic BCL2 and BCL-XL (44). BAX acts as pro-apoptotic effector mediating mitochondrial outer membrane permeabilization and is regulated by sequestration by anti-apoptotic BCL2 and BCL-XL (45). BIM acts as a sensor of growth factor deprivation and can activate BAX by its release from sequestration. Importantly, the pro-survival proteins BCL2 and BCL-XL play distinct roles in regulating survival of quiescent and activated lymphocytes, respectively (46). After *in vitro* culture with IL-2 we observed a decrease in BCL2 but increase in BCL-XL mRNA in activated Tregs which may be at least partially responsible for the enhanced cell proliferation and survival, although other mechanisms are also likely to also be active. Such an inverse correlation between levels of BCL2 and BCL-XL has been observed in a number of cancers (47). Notably, increased levels of BCL-XL but not BCL2 are observed in T cells in response to CD28 costimulation (48), in line with our observation in Tregs. The precise molecular mechanisms regulating apoptosis in Tregs are still under active investigation. When compared to conventional CD4^+^ T cells, freshly isolated human Tregs have been shown to be more sensitive to apoptosis than freshly isolated Teffs, but this is reversed after *in vitro* culture in the presence of CD3 and CD28 stimulation and exogenous IL-2 (49). Importantly, enhanced Treg survival correlated with the 2-fold increase in the expression of BCL2 and six-fold increase in BCL-XL expression (49). In mice, increased Bcl-xL expression has been demonstrated both in naturally-occurring (50) and induced (51) Tregs and ectopic co-expression of FoxP3 and Bcl-xL in CD4^+^ T cells induces regulatory cells with improved persistence and function *in vitro* and *in vivo* (52).

Regulation of BCL-XL is complex and is controlled on both transcriptional and post-transcriptional levels. miRNA Let-7c has been demonstrated to inhibit BCL-XL expression in hepatoma (30) and endothelial (29) cells. In our study increased BCL-XL expression in cultured Tregs negatively correlated with Let-7c expression, suggesting a similar control mechanism in Tregs. Let-7c miRNA belongs to the ubiquitously-expressed prototypical family of miRNAs (53). Targets for Let-7 include several genes involved in cell cycle and mitotic signaling such as HMGA2 (54), CDC25A and CDK6 (55), therefore its role in controlling Treg proliferation and survival is likely to be more complex and further studies are planned to explore this finding.

This is the first study to demonstrate and provide a potential mechanism for enhanced suppressive function in freshly isolated human Tregs following a short period of activation. This has important clinical implications, as it may provide a simple, efficient, rapid and cost-effective method for enhancing the suppressive capacity and survival of Treg cellular therapy.

## Ethics Statement

This study was carried out in accordance with the Helsinki Declaration and approved by the Oxfordshire Research Ethics Committee B (approval number 07/H0605/130) with written informed consent from all subjects.

The animal studies were carried out in strict accordance to the recommendations of the UK Animals (Scientific Procedures) Act of 1986. The protocol was approved by the University of Oxford Animal Care and Ethics Review committee and all procedures were carried under PPL P8869535A.

## Author Contributions

JH, FI, KM, RG, and GB performed the experiments. JH, FI, KM, and KW conceived and designed the experiments. JH and FI analyzed the data. JH, FI, and KW wrote the paper.

### Conflict of Interest Statement

The authors declare that the research was conducted in the absence of any commercial or financial relationships that could be construed as a potential conflict of interest.
